# Calcium Supplement Derived from *Gallus gallus domesticus* Promotes BMP-2/RUNX2/SMAD5 and Suppresses TRAP/RANK Expression through MAPK Signaling Activation

**DOI:** 10.3390/nu9050504

**Published:** 2017-05-17

**Authors:** Han Seok Yoo, Gyung-Ji Kim, Da Hye Song, Kang-Hyun Chung, Kwon-Jai Lee, Dong-Hee Kim, Jeung Hee An

**Affiliations:** 1 Department of Food Science and Technology, Seoul National University of Science & Technology, Seoul 01811, Korea; 0223yhs@naver.com (H.S.Y.); sdh5740@naver.com (D.H.S.); carl@seoultech.ac.kr (K.-H.C.); 2 Department of Chemical & Biomolecular Engineering, Sogang University, Seoul 04170, Korea; kgj8495@hanmail.net; 3 Department of Advanced Materials Engineering, Daejeon University, Daejeon 34520, Korea; jmul@ssu.ac.kr; 4 Department of Oriental Medicine, Daejeon University, Daejeon 34520, Korea; dhkim@dju.ac.kr; 5 Division of Food Bioscience, Konkuk University, Chungju 27478, Korea

**Keywords:** calcium, osteoporosis, differentiation, ovariectomy rat

## Abstract

The present study evaluated the effects of a calcium (Ca) supplement derived from *Gallus gallus domesticus* (GD) on breaking force, microarchitecture, osteogenic differentiation and osteoclast differentiation factor expression in vivo in Ca-deficient ovariectomized (OVX) rats. One percent of Ca supplement significantly improved Ca content and bone strength of the tibia. In micro-computed tomography analysis, 1% Ca supplement attenuated OVX- and low Ca-associated changes in bone mineral density, trabecular thickness, spacing and number. Moreover, 1% Ca-supplemented diet increased the expression of osteoblast differentiation marker genes, such as bone morphogenetic protein-2, Wnt3a, small mothers against decapentaplegic 1/5/8, runt-related transcription factor 2, osteocalcin and collagenase-1, while it decreased the expression of osteoclast differentiation genes, such as thrombospondin-related anonymous protein, cathepsin K and receptor activator of nuclear factor kappa B. Furthermore, 1% Ca-supplemented diet increased the levels of phosphorylated extracellular signal-regulated kinase and c-Jun N-terminal kinase. The increased expression of osteoblast differentiation marker genes and activation of mitogen-activated protein kinase signaling were associated with significant increases in trabecular bone volume, which plays an important role in the overall skeletal strength. Our results demonstrated that 1% Ca supplement inhibited osteoclastogenesis, stimulated osteoblastogenesis and restored bone loss in OVX rats.

## 1. Introduction

Osteoporosis, a major public health problem, is particularly prevalent in postmenopausal women. Genetics, deficient calcium (Ca) intake, cigarette smoking, excessive alcohol intake and reduction in estrogen levels are considered risk factors for osteoporosis [1]. Estrogen deficiency due to the cessation of ovarian function is an important contributor to bone loss in postmenopausal women [2]. The beneficial effects of estrogen replacement in the treatment of estrogen-deficiency-induced bone loss have been clearly established, but this therapy might enhance undesirable side effects include breast, endometrial cancers and ovarian in postmenopausal women [3,4,5]. Standard therapeutic drugs for osteoporosis, including antiresorptive drugs, such as bisphosphonate, osteocalcin and estrogen, do not significantly stimulate new bone synthesis [6]. Therefore, new agents for improving bone formation are needed, and functional foods containing natural substances as alternatives to estrogen replacement therapy represent a new approach for the prevention of postmenopausal osteoporosis [1]. 

Generally, Ca deficiencies are associated with hypertension, arteriosclerosis, diabetes, neurodegenerative diseases, malignant tumors and degenerative joint diseases [7]. National and international guidelines suggest that the Recommended Dietary Allowance (RDA) of Ca is 1000–1200 mg/day for patients with osteoporosis [8]. Many postmenopausal women do not acquire the optimum amount of Ca from the diet and rely on bioavailable Ca supplements [9]. Therefore, exploring suitable Ca supplementation or naturally occurring substances, especially those of animal origin, may help to prevent osteoporosis without causing adverse effects [10]. Previous studies of Ca supplementation showed that the bones of Nile tilapia (*Oreochromis niloticus*) and mola (*Amblypharyngodon mola*) fish were good sources of Ca [11,12]. In addition, milk and marine organisms, oyster shell electrolysate and calcium-binding peptide derived from pepsinolytic hydrolysates of hoki are important sources of calcium in the human diet [13,14,15]. Marine algae exhibit osteoblast cell proliferation and mineralization effects [9]. Moreover, sea tangle (*Kjellemaniella crassifolia*) intake contributes to the bone calcium content and breaking force of femurs in growing female rats [16]. Ca promotes osteoblast to osteocyte differentiation, as indicated by the increased expression of osteocalcin and runt-related transcription factor 2 (RUNX2) through the extracellular signal-regulated kinase (ERK) signaling pathway. However, the role of bone morphogenetic protein-2 (BMP-2), small mothers against decapentaplegic (SMADs) and RUNX2 in osteoblastogenesis and osteoclast-associated signaling of thrombospondin-related anonymous protein (TRAP), cathepsin K and receptor activator of nuclear factor kappa B (RANK) are still unknown.

*Gallus gallus domesticus* (GD) is a common Korean indigenous chicken breed. It possesses unique morphological features, such as black fluffy head feathers, earlobes, pupils and four toes [17]. Previous studies of Ogolgye chickens reported on their physicochemical characteristics and storage and sensory properties [17,18]. In addition, oligopeptide powder from this chicken has beneficial antioxidant effects [19]. Moreover, the water extracts of GD promoted alkaline phosphatase (ALP) activity and bone mineralization and inhibited bone resorption [20]. However, studies investigating the effects of the Ca derived from bone of GD on bone formation via osteoblast differentiation have not been reported.

Our study investigated the effect of Ca supplement derived from GD in a Ca-deficient ovariectomized (OVX) animal model. Ca supplement derived from GD showed effects on BMP-2, osteocalcin, collagenase-1 (COL-1), RUNX2 and SMAD5. Our results provide new insights into osteoblastic differentiation induced by Ca supplement and confirm its possible utilization as a functional food and bone health supplement.

## 2. Materials and Methods

### 2.1. Preparation of Sample

*Gallus gallus* var. *domesticus* chickens were obtained from Jisan Plantation (Chungnam, Republic of Korea) and reared until they were 3 years old. Bone samples were used after separating the bone from the other structures. The procedure for the preparation of bone powder was as follows: bone sample was dried at 110 °C for 18 h and kept under a vacuum in a desiccator. After being dried, the bone was homogenized and sieved with a stainless steel mesh (0.15–0.42 mm), and only the fine particles were mixed into rat feed.

### 2.2. Animals and Diet

All of the experiments were performed with the approval of the Institutional Animal Care and Use Committee at Konkuk University (IACUC, Approval Number KU 15133). Forty-eight 5-week-old Wistar-rats (body weight 117 ± 7 g) were purchased from Doo Yeol Biotech (Seoul, Republic of Korea). Forty-eight 5-week-old Wistar rats (body weight 117 ± 7 g) were purchased from Doo Yeol Biotech (Seoul, Korea). Animals were housed in an air-conditioned room at 23 ± 1 °C, 55–60% relative humidity, a 12 h light/dark cycle (07:00 lights on, 19:00 lights off). After acclimatization for 1 week, rats were anesthetized with 2% isoflurane, and ovaries were removed bilaterally. A sham operation, during which the ovaries were only touched with forceps, was performed on the sham group. After a 2-week acclimation period, the sham and normal diet groups were fed a normal (0.6% Ca) diet (TD.97191), while the other groups were fed a low Ca (0.01%) diet (TD.95027). All rats had free access to distilled water. Rats were divided into four treatment groups (12 rats per group) as follows: (1) sham (normal diet); (2) low Ca (OVX + low Ca); (3) normal diet (OVX + normal diet); (4) 1% Ca (OVX + low Ca + 1% Ca); dietary-supplement data shown in Table 1. Food intake was recorded every 3 days, and body weights were measured weekly. After the 8-week feeding period, rats were sacrificed by lethal intraperitoneal ether overdose. For each animal, tibial bones were dissected and stored at −20 °C. Micro-CT and breaking force were performed in the left tibia of all rats. Samples were analyzed by RT-PCR and Western blotting. The right tibia was used for calcium content measurement and histology experiments. All experiments were conducted in triplicate.

### 2.3. Biochemical Analyses 

Serum estrogen levels were measured using commercial kits (Creative Diagnostics, Shirley, NY, USA).

### 2.4. Biomechanical Testing of Tibia

Destructive biomechanical testing was performed on thawed specimens using the texture analyzer (Model TA.XT Express; Stable Micro Systems, Godalming, UK), employing exponent lite express software (Stable Micro Systems). The wedge was fractured by a downward motion (3 mm/s) of a steel blade with a thickness of 30 mm. The maximum force (highest value in N) applied to break the wedge was used to quantify the firmness. The samples were thawed, and all measurements were carried out at room temperature (25 °C). The breaking force of the tibia was evaluated by three-point bending (maximum breaking force of failure when a load was applied in a perpendicular plane to the longitudinal axis of the tibia). All tests were conducted in the mid-diaphyseal region of the tibia.

### 2.5. Ca Content of Tibia

Ca in the tibia was quantified using a microwave digestion system (Multiwave 3000; Anton Paar, Graz, Austria) and inductively coupled plasma mass spectrometry (HP-4500; Hewlett-Packard, Avondale, PA, USA). All tests were performed following official methods; association of official analytical chemists (AOAC).

### 2.6. Micro-Computed Tomography

Micro-CT was performed in the left tibia of all rats. Imaging of the cortical and trabecular bones was performed using a micro-CT system (Inveon PET; Siemens Medical Solutions, Knoxville, TN, USA) with the following acquisition parameters: 80 kVp, 500 μA, 211-ms exposure time, 30.74 mm field of view and 60.04-μm pixel size. A global threshold value was set to binarize bone tissue from non-bone tissue. A global threshold was visually determined by two independent examiners (based on slice-wise 2D comparisons between the grey scale and segmented image of all samples). The region of interest was indicated in green color in the trabecular bone. The resulting images were evaluated using the Inveon acquisition workplace software (Siemens Medical Solutions). Parameters such as bone mineral density (BMD), bone surface area/bone volume (BSA/BV), bone volume/total volume (BV/TV), trabecular thickness (Tb.Th), trabecular spacing (Tb.Sp) and trabecular plate number (Tb.N) were calculated three-dimensionally from measurements of trabecular bone mass and its distribution. The results were reported according to the guidelines for assessment of bone microarchitecture of rodents using μCT [21].

### 2.7. Histology and Tartrate-Resistant Acid Phosphatase Staining

The cleaned tibias were fixed in 10% neutral buffered formalin solution for 2 days at 40 °C. Decalcification was achieved by treatment with 10% EDTA (pH 7.4) replaced daily, under stirring, for 10 days at 25 °C and washed in tap water for 4 h. The tibias were paraffin-embedded, sectioned at 4 μm and stained with hematoxylin and eosin (H&E) [22]. Sections were stained after being mounted and observed for histopathological changes on the slides. Tartrate-resistant acid phosphatase (TRAP) and nuclear staining were performed according to the leukocyte acid phosphatase assay kit (Sigma Chemical Co., St. Louis, MO, USA) instructions. The specimens were examined using a Nikon Eclipse TS100 microscope (Nikon, Tokyo, Japan) at 200× magnification, and the results were analyzed using OptiView image analysis software (Korea Lab Tech, Seongnam, Korea).

### 2.8. Reverse Transcription-Polymerase Chain Reaction Assay

The total RNA was extracted from the tibia using TRIzol reagent (Thermo Fisher Scientific Inc., Waltham, MA, USA). For each reaction, 1 μg of total RNA was reverse-transcribed to cDNA using SuperScript III Reverse Transcriptase (Invitrogen, Carlsbad, CA, USA). The obtained cDNA was used to determine the tibia mRNA levels of BMP-2, RUNX2, Wnt3a, osteocalcin, COL-1, TRAP, cathepsin K and RANK using Taq DNA polymerase (Kapa Biosystems, London, UK). Glyceraldehyde 3-phosphate dehydrogenase (GAPDH) was used as an internal control. Primer sequences were as follows: GAPDH: 5′-AACTCCCATTCCACCTT-3′, 5′-GAGGGCCTCTCTCTTGCTCT-3′, BMP-2: 5′-AAGGCACCCTTTGTATGTGGACT-3′, 5′-CATGCCTTAGGGATTTTGGA-3′, RUNX2: 5′-TCCAGCCACCTTCACTTACAC-3′, 5′-GCGTCAACACCATCATTCTG-3′, Wnt3a: 5′-TCCGACTCTTGGCAGAACTT-3′, 5′-AATGGAATAGGTCCCGAACA-3′, osteocalcin: 5′-AGCTCAACCCCAATTGTGAC-3′, 5′-AGCTGTGCCGTCCATACTTT-3′, COL-1: 5′-TTGACCCTAACCAAGGATGC-3′, 5′-CACCCCTTCTGCGTTGTATT-3′, TRAP: 5′-CGCCAGAACCGTGCAGA-3′, 5′-TCAGGCTGCTGGCTGAC-3′, cathepsin K: 5′-CCCAGACTCCATCGACTATCG-3′, 5′-CTGTACCCTCTGCACTTAGCTGCC-3′, RANK: 5′-GTGACTCTCCAGGTCACTCC-3′, 5′-GGCAGACACACACTGTCG-3′. PCR was initiated at 95 °C for 3 min followed by 30 cycles at 95 °C for 30 s and 50–60 °C for 30 s. The number of cycles and annealing temperature for each primer pair were optimized. A final extension at 72 °C for 10 min was conducted. The PCR products were detected by 1.2% agarose gel electrophoresis with ethidium bromide and visualized using a UV transilluminator Genosens 1500 system (CLINX, Shanghai, China). Relative expression was quantified using Image J software (NIH, Bethesda, Rockville, MD, USA) and calculated compared to GAPDH. 

### 2.9. Western Blot Analysis

Tibial bone homogenates were lysed in lysis buffer containing protease inhibitor (Roche, Mannheim, Germany) and centrifuged at 10,000× *g* for 10 min at 4 °C. The total protein levels were determined using a Bio-Rad protein kit (Bio-Rad, Hercules, CA, USA). The proteins were subjected to 12% sodium dodecyl sulfate-polyacrylamide gel electrophoresis and transferred onto Immobilon-P transfer membranes (Millipore Co., Bedford, MA, USA), which were blocked with 5% bovine serum albumin prior to incubation with a specific primary antibody against BMP-2 (Abcam, Cambridge, UK), RUNX2 (Abcam), p-SMAD1/5/8 (Santa Cruz Biotechnology, Inc., Santa Cruz, TX, USA), Wnt3a (Abcam), osteocalcin (Abcam), COL-1 (Abcam), phosphorylated serine/threonine kinase (p-AKT) (Cell Signaling Technology, Danvers, MA, USA), p-ERK (Cell Signaling Technology), p38 (Cell Signaling Technology) and β-actin (Cell Signaling Technology) followed by goat anti-rabbit IgG (H + L) horseradish peroxidase (HRP)-conjugated secondary antibody (Zymax, San Francisco, CA, USA). The antigen-antibody complexes were visualized by enhanced chemiluminescence. Densitometric analysis of the signal was performed using a C-DiGit Blot Scanner (Li-COR Inc., Lincoln, NE, USA). Relative expression was quantified using Image J (NIH, Bethesda, Rockville, MD, USA) and compared to β-actin.

### 2.10. Immunohistochemical Stain

Tibia sections 4 µm thick were initially submitted to deparaffinization in serial concentrations of xylene and alcohol for 5 min, with subsequent recovery of antigenic sites on steam fluent (pot value) for 30 min. The slides were washed twice for 5 min with 1× phosphate-buffered saline (PBS), and then endogenous peroxidase blocking was carried out by immersion in hydrogen peroxide at 0.3% for 30 min at 25 °C. Sections were incubated with 10% goat (for polyclonal antibodies) serum for 30 min before overnight incubation at 4 °C followed by incubation with the appropriate specific primary antibody (BMP-2 (Abcam), RUNX2 (Abcam), Wnt3a (Abcam), osteocalcin (Abcam) or COL-1 (Abcam)). Samples were then incubated with biotinylated goat anti-rabbit IgG (H + L) HRP-conjugated (or goat anti-mouse IgG (H + L) HRP-conjugated) antibodies (Zymax). The sections were incubated with 3,3′diaminobenzidine (DAB; Sigma Chemical Co.) for 15 min at 25 °C. The sections were then counterstained with hematoxylin solution for 20 s, fixed in alcohol-xylene series and mounted with cover slip and mounting medium Permount (Thermo Fisher Scientific). Negative controls were incubated with normal goat IgG (Zymax, San Francisco, CA, USA) instead of the primary antibody. The specimens were examined using a Nikon Eclipse TS100 microscope (Nikon) at 200× magnification, and the results were analyzed using OptiView image analysis software (Korea Lab Tech).

### 2.11. Statistical Analysis 

Statistical analysis was performed using SPSS 18.0 (SPSS Inc., Chicago, IL, USA). Averages and standard deviations were calculated, and differences between groups were assessed by the analysis of variance (ANOVA) method and Duncan’s multiple range test. A difference was considered significant if *p* < 0.05.

## 3. Results

### 3.1. Serum Estrogen Concentrations of Tibia

**Figure 1:**
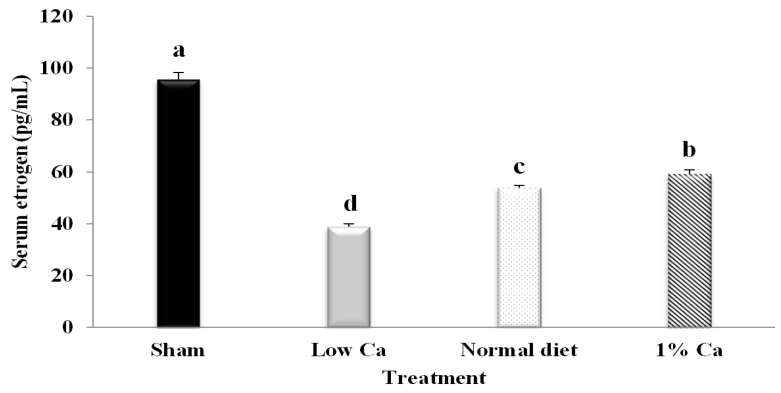
In vivo serum estrogen concentrations. Results are expressed as the mean ± standard deviation (SD). Values not sharing a common superscript (**a**, **b**, **c** and **d**) differed significantly (Duncan’s multiple range test).

Serum estrogen levels were measured to determine the effect of estrogen on bone mass. Normal physiological estrogen concentrations were determined using serum samples from OVX rats. Serum estrogen concentrations were significantly decreased in the low Ca group compared to in the other study groups (Figure 1). However, evaluation of the normal diet and 1% Ca groups showed that estrogen levels increased by 38.7% and 52.8%, respectively, compared to that in the low Ca group. These data indicate that 1% Ca restored serum estrogen in OVX rats.

### 3.2. Feed Efficiency Ratio

The food intake, body weight gain and food efficiency ratio (FER) did not differ significantly among the experimental groups. However, the weight gain of low Ca group was lower than that of the other groups (Table 2).

### 3.3. Tibia Bone Strength and Ca Content of Tibia

**Figure 2:**
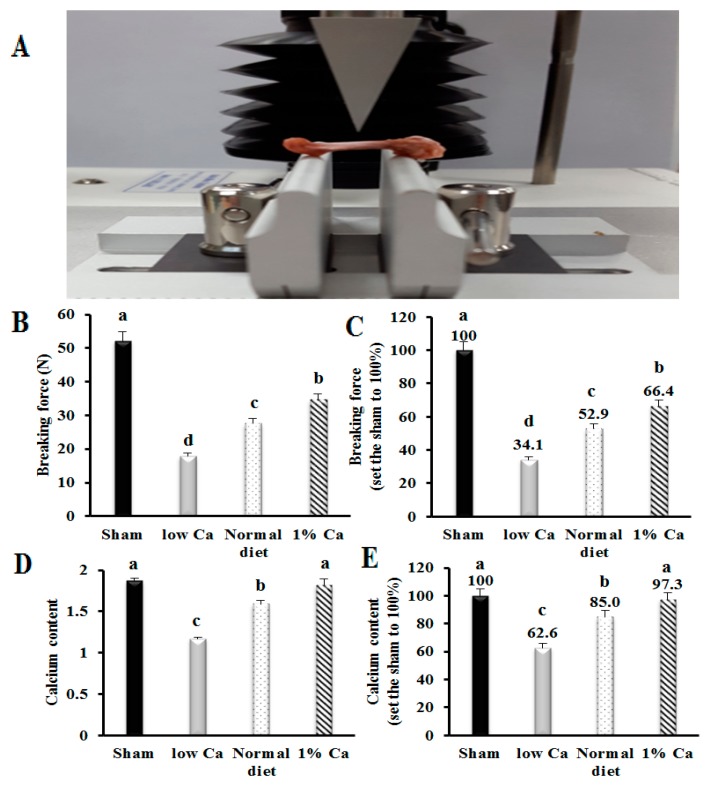
Effects of breaking force analysis on rat tibias. (**A**) Details of breaking force analysis; (**B**) Ratio of breaking force required in rat tibias; (**C**) The value of the bone strength when sham was set to 100%; (**D**) Tibial calcium levels in the indicated rats; (**E**) The value of the tibial calcium levels when sham was set to 100%. Results are expressed as the mean ± standard deviation (SD). Values not sharing a common superscript (**a**, **b**, **c** and **d**) differed significantly (Duncan’s multiple range test).

In order to investigate the effects of Ca supplement on bone strength, we conducted breaking force tests using a texture analyzer (Figure 2A). The breaking force of the low Ca group was significantly lower than that in the sham group. Assuming that the breaking force of the sham group was 100%, the low Ca, normal diet and 1% Ca groups breaking forces were 34.1%, 52.9% and 66.4%, respectively. The breaking force of the low Ca group decreased 65.9% compared to the sham group. In addition, breaking force of the normal diet and 1% Ca groups increased 18.8% and 32.3% compared to low Ca group, respectively (*p* < 0.05) (Figure 2B,C). Thus, 1% Ca supplement feed improved tibial breaking force compared to the low Ca group. These results suggested that the increase of breaking force depended on the increase of Ca content. 

Assuming that the Ca content in the Sham group was 100%, the tibia Ca content of the 1% Ca group increased 34.7% and 12.3% compared to low Ca and normal diet groups, respectively. Furthermore, there were no significant difference between sham and 1% Ca groups (Figure 2D,E). Thus, these findings proved that the dietary supplementation of 1% Ca increased Ca contents of the tibia. 

### 3.4. Effects on Bone Morphometric Parameters

**Figure 3:**
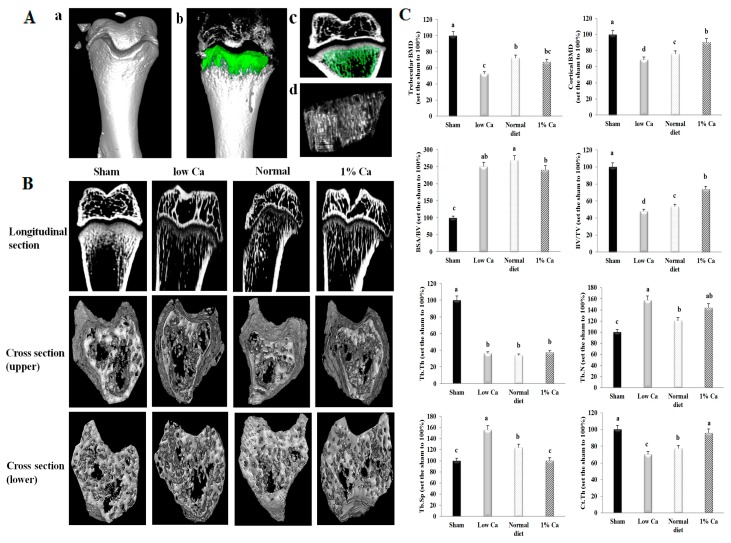
Tibial bone microstructure analyses in rats. (**A**) Process used to analyze trabecular and cortical bone and resulting measurements. a indicates the three-dimensional structure of the tibia, while b shows the three-dimensional green-colored area used to measure tibial parameters. The green-colored area in c indicates the longitudinal section of b. d shows the image of isosurfaces, taken from the green-colored region indicated in b and c. (**B**) Longitudinal section and cross-section of the trabecular in sham, low Ca, normal diet, and 1% Ca groups. (**C**) The micro-CT scanner parameter of trabecular BMD, cortical BMD, tibia bone surface area, volume, thickness, spacing, number and cortical thickness in sham, low Ca, normal diet, and 1% Ca groups. Results are expressed as the means ± standard deviation (SD). Values not sharing a common superscript (**a**, **b**, **c** and **d**) differed significantly (Duncan’s multiple range test).

We performed ex vivo micro-CT after eight weeks on all animals, and tibial architecture was investigated using maximum intensity projection images (Figure 3). Figure 3A illustrates the process used to analyze trabecular and cortical bone and the resulting measurements. Figure 3a indicates the three-dimensional (3D) structure of the tibia, and Figure 3b shows the 3D green-colored area used to measure tibial parameters. The green-colored area of Figure 3c indicates the longitudinal section of Figure 3b. Figure 3d shows the image of isosurfaces taken from the green-colored region indicated in Figure 3b,c. Micro-CT scanning and analysis revealed that the rat tibial bones were severely affected by ovariectomy. The scanning results showed that in the low Ca group bone mass decreased compared to the sham group. Micro-CT scanning also confirmed that the 1% Ca-supplemented diet showed induced remission of bone loss after eight weeks in OVX rats (Figure 3B). Longitudinal sections of tibias from the low Ca group showed central spaces in the trabecular bone. In addition, upper and lower tibial cross-sections from the low Ca group showed large spaces within the marrow. However, longitudinal and cross-sectional analyses of sham, normal diet and 1% Ca-supplemented groups showed that the bone marrow space was more occupied compared to the low Ca group.

The bone structure of 1% Ca groups was distributed relatively uniformly to form a well-connected network compared to the low Ca group (Figure 3B). Micro-CT analysis of the tibia metaphysis was used to calculate trabecular BMD, cortical BMD, BSA/BV, BV/TV, Tb.Th, Tb.Sp, Tb.N and Ct.Th. Trabecular BMD values of sham, low Ca, normal diet and 1% Ca rats were 291, 153, 210 and 196 mg/cm^3^, respectively. In addition, the cortical BMD values of sham, low Ca, normal diet and 1% Ca rats were 1069, 734, 811 and 966 mg/cm^3^, respectively. Analysis of data indicated that low Ca feed decreased BV/TV, Tb.Th and Ct.Th. In contrast, low Ca feed increased BSA/BV, Tb.N and Tb.Sp. In addition, the 1% Ca supplement from GD increased BV/TV and Ct.Th by 53.5% and 36.5% compared to the low Ca group, respectively. Furthermore, BSA/BV, Tb.N and Tb.Sp decreased 13.8%, 15% and 54.9% compared to the low Ca group. However, Tb.Th was no significantly different in low Ca and 1% Ca groups (Figure 3C). These data indicated that 1% Ca supplementation was more effective in relieving osteoporosis than normal diets, when osteoporosis is already induced. 

### 3.5. Expression of Osteoblastogenesis Marker Genes

**Figure 4:**
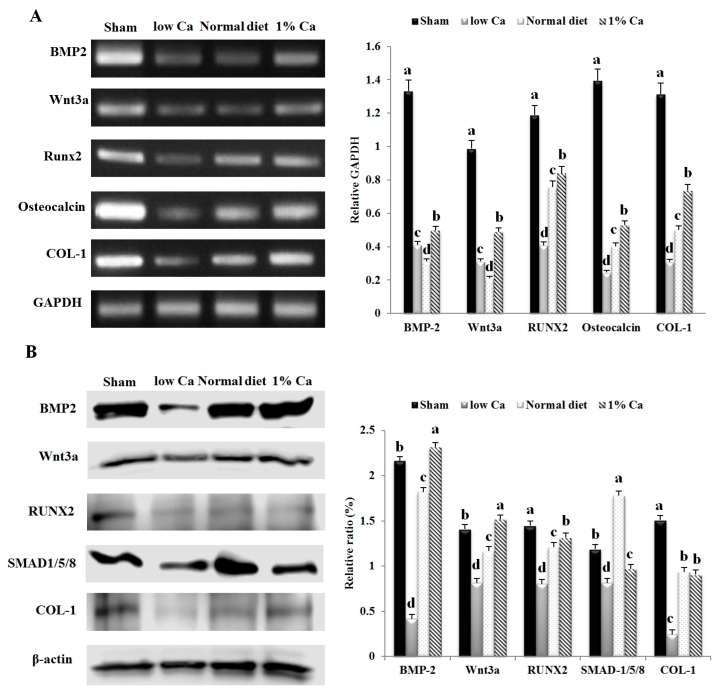
The expression of mRNA and protein levels of molecules involved in bone metabolism in sham, low Ca, normal diet, and 1% Ca groups. (**A**) The mRNA expression of bone morphogenetic protein-2 (BMP-2), Wnt3a, runt-related transcription factor 2 (RUNX2), osteocalcin and collagenase-1 (COL-1) in sham, low Ca, normal diet and 1% Ca groups; (**B**) The protein expression of BMP-2, Wnt3a, RUNX2, small mothers against decapentaplegic (SMAD)1/5/8, and COL-1 in sham, low Ca, normal diet and 1% Ca groups. Results are expressed as the means ± standard deviation (SD). Values not sharing a common superscript (**a**, **b**, **c**, and **d**) differed significantly (Duncan’s multiple range test).

We examined the impact of 1% Ca supplementation on bone metabolism indicators, including BMP-2, Wnt3a, SMAD1/5/8, RUNX2, osteocalcin and COL-1, by RT-PCR and Western blot analysis (calculated compared to GAPDH and β-actin), as shown in Figure 4. The mRNA expression level of BMP-2, Wnt3a, RUNX2, osteocalcin and COL-1 of the 1% Ca group was significantly upregulated compared to the low Ca group. In particular, the mRNA expression level of COL-1 was 2.4-fold higher than that of the low Ca group. Furthermore, in 1% Ca group, the expression level of Wnt3a, RUNX2 and osteocalcin significantly increased by 1.5-, 2.0- and 2.2-fold compared to that of the low Ca group, respectively (Figure 4A). As shown in Figure 4B, in the 1% Ca group protein expression of BMP-2, Wnt3a, RUNX2, SMAD1/5/8 and COL-1 was enhanced compared to that in the low Ca group. Although the mRNA expression of BMP-2 showed a low rising level, in the 1% Ca group, the protein expression of BMP-2 was dramatically enhanced by 5.6-fold. Moreover, Wnt3a, RUNX2, SMAD1/5/8 and COL-1 levels in the 1% Ca group were enhanced 1.8-, 1.6-, 1.2- and 4.5-fold compared to those in the low Ca group, respectively (Figure 4). These results suggested that, in vivo, 1% Ca supplement promoted the expression of genes involved in osteoblastogenesis.

**Figure 5:**
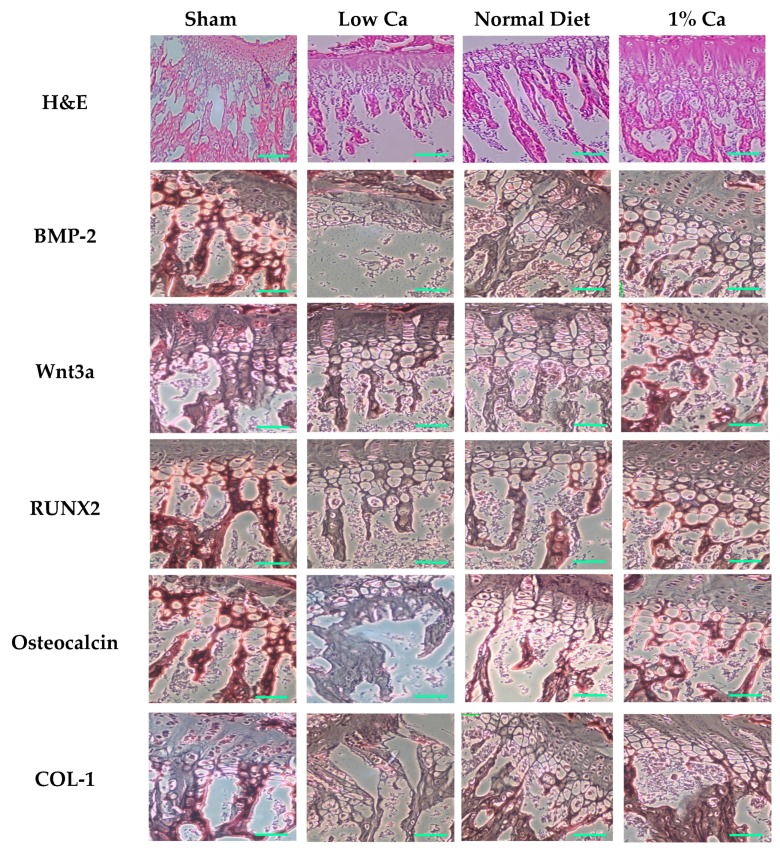
Hematoxylin and eosin (H&E) and immunohistochemical staining image of bone morphogenetic protein-2 (BMP-2), Wnt3a, runt-related transcription factor 2 (RUNX2), osteocalcin and collagenase-1 (COL-1) in sham, low Ca, normal diet, and 1% Ca groups (scale bar: 40 µm).

In order to assess the development of osteoporosis, we performed H&E and immunohistochemical staining. The bone trabeculae of rats in the low Ca group were arranged sparsely in a disorderly manner and indicated apparent signs of resorption. In contrast, the trabecular bone arrangement in the 1% Ca group was more regular (Figure 5). In addition, all osteogenesis-related proteins (BMP-2, Wnt3a, RUNX2, osteocalcin and COL-1) showed decreased expression and mild staining pattern in the low Ca group, while the sham, normal diet and 1% Ca groups showed more intense staining. These findings suggest that 1% Ca supplementation upregulated specific genes involved in osteoblastogenesis, such as BMP-2, Wnt3a, RUNX2, SMAD1/5/8, osteocalcin and COL-1.

### 3.6. Effects of Ca on the Expression of Osteoclast Specific Gene Markers

**Figure 6:**
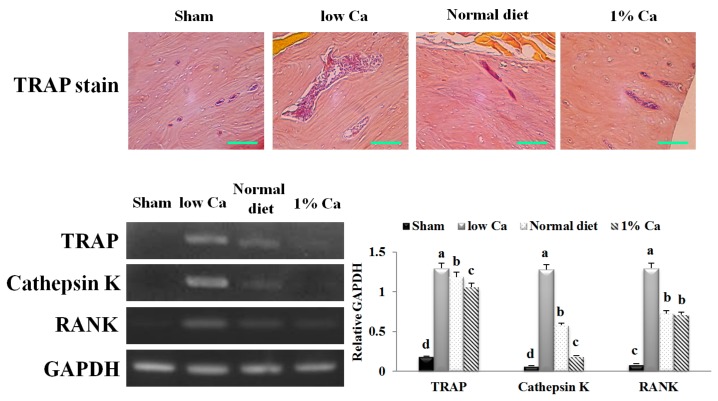
Tartrate-resistant acid phosphatase (TRAP) staining (scale bar: 40 µm) and mRNA expression of TRAP, cathepsin K and receptor activator of nuclear factor kappa B (RANK) in sham, low Ca, normal diet and 1% Ca groups. Results are expressed as the means ± standard deviation (SD). Values not sharing a common superscript (**a**, **b**, **c**, and **d**) differed significantly (Duncan’s multiple range test).

To assess osteoclast development, we performed TRAP staining. Multinucleated TRAP-positive osteoclasts were detected in the subchondral bone marrow space of the tibia at eight weeks after surgery (Figure 6). However, in the low Ca group, TRAP-positive osteoclasts were primarily detected in the tibial subchondral bone marrow space, in contrast to other groups (Figure 6). We observed a highly significant decrease in TRAP expression in the 1% Ca group compared to in the low Ca and normal diet groups. We observed a highly significant decrease in TRAP expression in the 1% Ca group compared to that in low Ca and normal diet groups. The expression level of cathepsin K in the 1% Ca group was 6.85- and 3.05-fold lower than that observed in low Ca and normal diet groups, respectively. In addition, the RANK expression level in the 1% Ca group was 1.84-fold lower than that observed in the low Ca group, but was not significantly different from the normal diet group (Figure 6). These results suggested that, in vivo, 1% Ca supplement suppressed the relative mRNA expression of genes involved in osteoclastogenesis.

### 3.7. Effects of 1% Ca Supplement on Phosphorylation of AKT, ERK and p38 MAPKs

**Figure 7:**
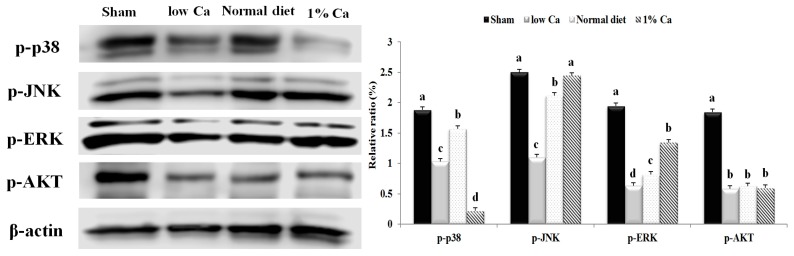
The expression level of phosphorylated p38, c-Jun N-terminal kinase (JNK), extracellular signal-regulated kinase (ERK) and serine/threonine kinase (AKT) in sham, low Ca, normal diet and 1% Ca groups. Results are expressed as the means ± standard deviation (SD). Values not sharing a common superscript (**a**, **b**, **c** and **d**) differed significantly (Duncan’s multiple range test).

Several studies [23,24,25] have indicated that the MAPK/ERK pathway is essential for the regulation of osteoblast differentiation and maturation. Therefore, we examined the effects of 1% Ca supplement on the activation of phosphorylated AKT, p38, ERK and JNK proteins in tibia. As shown in Figure 7, the activation level of p-ERK and p-JNK significantly increased in the 1% Ca group and significantly decreased in the low Ca group. In particular, the activation level of p-JNK in the 1% Ca group increased 2.18-fold compared to that of the low Ca group. However, *p*-p-38 and p-AKT showed no significant difference (Figure 7). These data suggested that p-ERK and p-AKT signaling pathways were activated in cells treated with 1% Ca. Thus, these results suggested that 1% Ca supplement activated ERK and JNK signaling pathways, leading to osteoblast differentiation and maturation. 

## 4. Discussion

Previous studies showed that the water extract from GD promotes osteoblast differentiation and inhibits osteoclast differentiation [20]. In the present study, we investigated the effects of Ca supplementation derived from GD on breaking force, bone microarchitecture and bone metabolism genes using a Ca-deficient OVX rat model. Based on our experiments, the Ca supplement derived from GD may be an effective supplement for bone health and osteoporosis prevention. Our study is the first report on regulatory effects of Ca supplement derived from GD that upregulates osteogenesis and inhibits osteoclastogenesis through ERK and JNK pathway activation.

Moreover, we showed that OVX surgery caused bone loss. Furthermore, the low Ca diet was administered, and Ca content and bone strength of rat tibia were dramatically reduced. In contrast, the Ca content and breaking force of tibias in the 1% Ca group were 34.8% and 32.3% higher than those in the low Ca group, respectively. We observed a similar pattern to that reported in previous studies where nano-calcium-supplemented milk increased the bone stiffness and Ca content in OVX rats [13]. Ingestion of hake fish bone may be helpful to increase bone Ca content, as well as the breaking force of rat bones. Therefore, intake of 1% Ca supplement derived from GD may improve tibial Ca content and breaking force. 

Traditionally, bone microarchitectural analyses have been conducted using histological sections (two-dimensional measurements) [26,27]. However, micro-CT has the advantage of providing higher resolution and is a non-destructive, easy and rapid method [28]. The recent improvements in spatial resolution and throughput of 3D imaging led to identifying and measuring bone parameters associated with osteoporosis. High-resolution micro-CT analyses revealed that the tibia from the low Ca group contained lesser trabecular bone in the marrow area compared to that from the other groups. In some sections, there was no bone and a large space in the trabecular structure. In addition, the low Ca group displayed loss of BV/TV, Tb.Th, Tb.N and Ct.th and an increase in BSA/BV and Tb.Sp, indicating the development of osteopenia (Figure 3 and Figure 4). However, in the same sections in sham, normal diet and 1% Ca groups, the bone marrow space was more occupied by the trabecular bone structure, which was distributed in a relatively uniform manner to form a well-connected network (Figure 3). We also observed that 1% Ca supplement improved the BV/TV of the distal femur. It has been shown that salmon bone powder improved the BV/TV of OVX rats [29,30]. The observed increases in BV/TV, Tb.Th, Tb.N and Ct.Th, and the decrease in BSA/BV and Tb.Sp indicated an improved overall bone strength and tibial trabecular bone density. Micro-CT analyses indicated that 1% Ca supplement was able to attenuate OVX-induced osteoporosis and prevent the detrimental effects of a low Ca diet.

**Figure 8:**
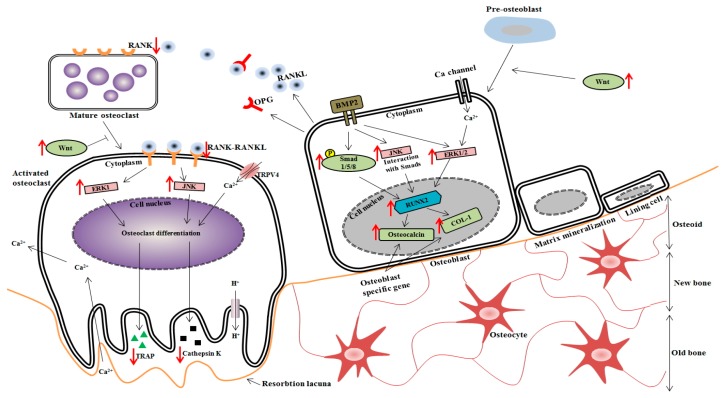
Scheme of Ca signaling derived from *Gallus gallus domesticus* (GD) on osteoblastogenesis and osteoclastogenesis.

In osteoblast differentiation [31,32], the genes for BMP-2, COL-1 and osteocalcin are involved in the formation, metabolism and regeneration of bone [33]. BMP-2, an important growth factor, modulates osteoblast differentiation by stimulating osteoblast-related transcriptional factors, including RUNX2 [34]. In addition, SMAD1 and the closely related SMAD5 and eight specifically mediate BMP-2 responses, such as osteoblastic differentiation of precursor cell lines [35]. Especially, osteocalcin has the ability to bind hydroxyapatite and Ca and is highly expressed in growing skeletal tissue. It plays an important role in the mineralization or bone turnover process or both [36]. The present study demonstrated that the 1% Ca-supplemented diet upregulated the expression of genes, such as BMP-2, Wnt3a, SMAD1/5/8, RUNX2, osteocalcin and COL-1 compared to the low Ca group. These results were in agreement with those of previous studies showing that Ca-treated cells and elevated concentrations of extracellular Ca induced an increase in osteocalcin mRNA expression [37,38]. In addition, Ca sources such as coral sand increased RUNX2 and COL-1 mRNA expression [39]. Moreover, Sr-Ca co-administration upregulated RUNX2 mRNA expression [40]. These results provide important insights into how the downstream components of the BMP-2 signaling pathways, which we have identified as RUNX2 and receptor-activated SMADs and induce osteoblast differentiation in OVX rat (Figure 8). Thus, 1% Ca supplement promoted osteoblast differentiation and induced the BMP/SMADs/RUNX2 signaling pathway.

TRAP is an iron-binding protein that is highly expressed in osteoclasts and induced in differentiation of osteoclasts [41]. Cathepsin K is a recently identified lysosomal cysteine proteinase. It is abundant in osteoclasts, where it plays a pivotal role in the remodeling and resorption of bone [42]. Monocytes differentiate into osteoclasts through stimulation of RANK. Many downstream effectors of RANK play a positive role in osteoclastogenesis [43]. Decreases in TRAP, cathepsin K and RANK activity have been used as valuable indicators of an osteoclast. The present study demonstrated that the 1% Ca-supplemented diet decreased TRAP, cathepsin K and RANK mRNA expression compared to the low Ca group. These results were in agreement with those of previous studies showing that the presence of high extracellular concentrations of Ca and 1,25-dihydroxyvitamin D3 decreased mRNA expression of cathepsin K [44]. Based on these findings, it is suggested that 1% Ca supplement may not only have inhibited effect on the expression of RANK at early stage, but also could repress TRAP and cathepsin K osteoclastogenesis at a late stage (Figure 8). 

It is well known that MAPK signaling pathways including ERK, P38 and JNK signaling pathways are crucial for the regulation of cell proliferation, osteoblast differentiation and skeletal development. Previous studies have shown that the ERK signaling pathway plays a pivotal role in mediating the collagen or collagen-related peptide-induced osteogenic differentiation of osteoblast precursors or mesenchymal stem cells [45,46,47,48]. The present study demonstrated that the 1% Ca-supplemented diet promoted the activation of p-ERK and p-JNK signaling. Previous reports demonstrated that Ca is important in determining the specificity of the ERK cascade [49]. Another study reported that Ca-containing crystals enhanced the phosphorylation level of ERK, but did not change the phosphorylation levels of AKT and JNK [50]. In addition, Ca-, Mg- and Si-containing akermanite bioceramics (Ca_2_MgSi_2_O_7_) may activate p38, ERK and AKT signaling pathways [51]. However, another study reported that akermanite bioceramics stimulated osteogenic differentiation via activation of the ERK pathway, while there was no detectable activation of the p38 signaling pathway [52,53]. Consistent with these observations, the present study found that the 1% Ca group showed a significant increase in the ratio of phosphorylated ERK and JNK protein expression. However, our study showed that osteoblast differentiation was stimulated by activation of ERK and JNK, but not by activation of p38. Based on these findings, BMP-2 can activate ERK and JNK in OVX rats and provide evidence that these MAP kinases have distinct roles in regulating RUNX2, osteocalcin and COL-1 (Figure 8). In osteoclast differentiation, RANKL binds to RANK in osteoclast precursor and differentiating osteoclasts cells, resulting in activation of various intracellular signaling pathways involving ERK and JNK [39]. Thus, our study demonstrated that 1% Ca supplement was able to promote osteoblast differentiation and via ERK and JNK activation and hence bring about the upregulation of BMP-2, SMAD1/5/8, RUNX2, osteocalcin and COL-1. Furthermore, it inhibited osteoclast differentiation genes (RANK, TRAP and cathepsin K) via ERK and JNK activation.

## 5. Conclusions

For the first time, the present study demonstrated that 1% Ca supplement derived from GD enhanced breaking force, microarchitecture and expression of genes related with osteoblast differentiation. Furthermore, these Ca extracts increased the expression levels of BMP-2, Wnt3a, SMAD5, RUNX2, osteocalcin and COL-1 in an in vivo model of osteoporosis, while they decreased TRAP, cathepsin K and RANK expression levels. TRAP, cathepsin K and RANK also stimulated the ERK and JNK signaling pathways. These changes were associated with significant increases in BMD and trabecular bone volume, which play an important role in overall skeletal strength. These findings provide proof that 1% Ca supplement derived from GD may help to delineate the potential of collagen for the treatment of bone metabolism disorders.

## Figures and Tables

**Table 1:** Composition of the experimental diets.

Composition	Sham	Low Ca	Normal Diet	1% Ca
Casein (g/kg)	200.0	200.0	200.0	200.0
L-Cystine (g/kg)	3.0	3.0	3.0	3.0
Sucrose (g/kg)	334.288	334.288	334.288	334.288
Corn Starch (g/kg)	313.0	320.0	313.0	320.0
Soybean Oil (g/kg)	60.0	60.0	60.0	60.0
Cellulose (g/kg)	40.0	40.0	40.0	40.0
Mineral Mix (g/kg) ^a^	13.37	13.37	13.37	13.37
Potassium Phosphate, monobasic (g/kg)	11.43	11.43	11.43	11.43
Vitamin Mix (g/kg) ^b^	10.0	10.0	10.0	10.0
Calcium (%)	0.6	0.01	0.6	1.0
P (%)	0.4	0.4	0.4	0.4

^a^ (NaCl: 193.7325, C_6_H_7_K_3_O_8_: 575.9615, K_2_SO_4_: 136.1363, MgO: 62.8322, MnCO_3_: 9.163, C_6_H_5_FeO_7_: 15.708, ZnCO_3_: 4.1888, CuCO_3_: 0.7854, KIO_3_: 0.0262, Na_2_SeO_3_·5H_2_O: 0.0262, CrK(SO_4_)_2_·12H_2_O: 1.4399). ^b^ (p-Aminobenzoic Acid: 11.0132, Vitamin C, ascorbic acid, coated (97.5%): 101.6604, Biotin: 0.0441, Vitamin B_12_ (0.1% in mannitol): 2.9736, Calcium Pantothenate: 6.6079, Choline Dihydrogen Citrate: 349.6916, Folic Acid: 0.1982, Inositol: 11.0132, Vitamin K3, menadione: 4.9559, Niacin: 9.9119, Pyridoxine HCl: 2.2026, Riboflavin: 2.2026, Thiamin (81%): 2.2026, Vitamin A Palmitate (500,000 IU/g): 3.9648, Vitamin D3, cholecalciferol (500,000 IU/g): 0.4405, Vitamin E, DL-alpha tocopheryl acetate (500 IU/g): 24.2291, Corn Starch: 466.6878).

**Table 2:** Bodyweight and food intake.

	Sham	Low Ca	Normal Diet	1% Ca
Feed intake (g/day)	33.60 ± 4.19 ^a^	34.80 ± 3.84 ^a^	33.20 ± 3.64 ^a^	33.30 ± 4.74 ^a^
Body weight (g)				
Initial	117.33 ± 8.4 ^a^	118.75 ± 7.68 ^a^	118.50 ± 7.0 ^a^	113.66 ± 5.8 ^a^
Final	339.75 ± 16.34 ^a^	324.25 ± 30.89 ^a^	338.25 ± 32.34 ^a^	320.33 ± 50.58 ^a^
Body weight gain (g/week)	32.90 ± 2.24 ^a^	30.81 ± 3.21 ^a^	33.14 ± 3.97 ^a^	32.59 ± 6.54 ^a^
FER *	0.14 ± 0.02 ^a^	0.13 ± 0.01 ^a^	0.14 ± 0.02 ^a^	0.13 ± 0.03 ^a^

* Body weight gain (g/week)/feed intake (g/week). ^a^ (NaCl: 193.7325, C_6_H_7_K_3_O_8_: 575.9615, K_2_SO_4_: 136.1363, MgO: 62.8322, MnCO_3_: 9.163, C_6_H_5_FeO_7_: 15.708, ZnCO_3_: 4.1888, CuCO_3_: 0.7854, KIO_3_: 0.0262, Na_2_SeO_3_·5H_2_O: 0.0262, CrK(SO_4_)_2_·12H_2_O: 1.4399).
